# The Real Impact of the Coronavirus Disease 2019 (covid-19) on the Pregnancy Outcome

**DOI:** 10.1055/s-0040-1712942

**Published:** 2020-05

**Authors:** Ana Katherine Gonçalves

**Affiliations:** 1 Universidade Federal do Rio Grande do Norte, Natal, RN, Brazil

**Keywords:** COVID-19, SARS-CoV-2, pregnant, gravidity, maternal-fetal, COVID-19, SARS-CoV-2, gravidez, gravidade, maternal-fetal

## Abstract

The COVID-19 outbreak is increasing around the world in the number of cases, deaths, and affected countries. Currently, the knowledge regarding the clinical impact of COVID-19 on maternal, fetal, and placental aspects of pregnancy is minimal. Although the elderly and men were the most affected population, in previous situations, such as the 2009 H1N1 influenza pandemic and the Ebola epidemic, pregnant women were more likely to develop complications than nonpregnant women. There are unanswered questions specific to pregnant women, such as whether pregnant women are more severely affected and whether intrauterine transmission occurs. Additional information is needed to inform key decisions, such as whether pregnant health care workers should receive special consideration, whether to separate infected mothers and their newborns, and whether it is safe for infected women to breastfeed.

## Resumo

A COVID-19 está se disseminando em todo o mundo, expandindo o número de casos, mortes e países afetados. Atualmente, o conhecimento sobre o impacto da COVID-19 nos desfechos materno, fetal e placentário da gravidez é mínimo. A despeito de serem os homens e idosos a população mais afetada em situações anteriores, como a pandemia de influenza H1N1 de 2009 e a epidemia de Ebola, as gestantes foram mais propensas a desenvolver complicações do que as mulheres não grávidas. Em relação à gravidez, existem questões específicas a serem respondidas, tais como se as mulheres grávidas são mais gravemente afetadas e se ocorre transmissão intrauterina. São necessárias informações adicionais para embasar a tomada de decisões, tais como; se as profissionais de saúde grávidas devem receber atenção especial, se é necessário separar as mães infectadas dos seus recém-nascidos e se é seguro que as puérperas infectadas amamentem.

Dear Editor,

We read with great interest the recent publications about the COVID-19 outbreak. The most vulnerable individuals have already been identified. Initially, men and the elderly were more frequent among hospitalized cases, and children and pregnant women have rarely been reported. In the beginning, pregnant women are not more severely affected than the general population,^1^ although several questions remain about pregnant women and their newborns.

It is important to remember that during the 2009 H1N1 influenza pandemic, women were more likely to develop complications than nonpregnant women.^2^ Furthermore, during the previous Ebola epidemic, the CDC recommended that pregnant health care workers not care for patients with Ebola virus disease, given the severity of illness in mothers and their neonates, the high risk of transmission, and the challenges related to the required personal protective equipment for Ebola care.^3^

Additionally, the numbers of pregnant women reported having the same conditions (being of similar age to the population with COVID-19) have still been very low.^1^ Thus, additional information is critically needed to inform key decisions, such as whether pregnant health care workers should receive special consideration, whether to separate infected mothers and their newborns, and whether it is safe for infected women to breastfeed. There are other unanswered questions specific to pregnant women, such as whether pregnant women are more severely affected and whether intrauterine transmission occurs.

Liu et al^4^ reported a clinical course and outcome of pregnant patients with COVID-19 showing high complication rates. Most of the patients had to deliver by emergency cesarean (including one stillbirth) due to a variety of indications, and most had preterm delivery. In this study, one mother had multiple organ failure and was on life support with extracorporeal membrane oxygenation at the time of reporting.^4^ Chen et al^5^ reported a cesarean section rate of 100%, but no preterm birth or neonatal death. The cesarean section was performed since these women were symptomatic with COVID‐19 pneumonia in their 3^rd^ trimester. However, no maternal mortality has been reported so far by both studies.^4^ ^5^

Vertical transmission is always a concern.^6^ Zeng et al^7^ in a cohort demonstrated that 3 out of 33 infants (9%) presented early-onset SARS-CoV-2 infection, due to strict infection control and prevention procedures being implemented during the delivery, thus it is likely that the sources of SARS-CoV-2 in the upper respiratory tracts or anuses of the neonates were maternal in origin. Therefore, the latter study^7^ claims that it is crucial to screen pregnant women and implement strict infection control measures, quarantine the infected mothers, and closely monitor neonates at risk of COVID-19.

Definitely, more studies are needed to clarify other outcomes inherent to pregnancy, such as fetal-maternal morbidity and mortality, intrauterine growth restriction, arterial hypertension, renal failure, HELLP syndrome, miscarriage, and placenta previa.

Similar to previous emerging situations, we must base recommendations on the best medical evidence available now, recognizing that data are limited, and that recommendations could change in the future.

Until now, the safe way to prevent the spread of COVID-19 is to implement measures that we use every year to limit the spread of seasonal influenza, such as avoid contact with ill persons, avoid touching their face, wash hands frequently, disinfect contaminated surfaces, and stay home when sick. Prenatal clinics should ensure all pregnant women are screened for fever, and respiratory symptoms and symptomatic women should be isolated from healthy women and required to wear a mask. Additional measures, such as limiting visitors in delivery units and postpartum wards, should be adopted.^1^
